# Control of interjoint coordination during the swing phase of normal gait at different speeds

**DOI:** 10.1186/1743-0003-4-10

**Published:** 2007-04-27

**Authors:** Jonathan Shemmell, Jennifer Johansson, Vanessa Portra, Gerald L Gottlieb, James S Thomas, Daniel M Corcos

**Affiliations:** 1 Neuromuscular Research Center, Boston University, Boston, MA 02215, USA; 2 Department of Physical Medicine and Rehabilitation, Harvard Medical School, Spaulding Rehabilitation Hospital, Boston, MA 02114, USA; 3 School of Physical Therapy, Ohio University, Athens, OH 45701, USA; 4 Department of Movement Sciences, University of Illinois at Chicago, Chicago, IL 60612, USA; 5 Department of Bioengineering, University of Illinois at Chicago, Chicago, IL 60612 ,USA; 6 Department of Physical Therapy, University of Illinois at Chicago, Chicago, IL 60612, USA; 7 Department of Neurological Sciences, Rush Medical College, Chicago, IL 60612, USA

## Abstract

**Background:**

It has been suggested that the control of unconstrained movements is simplified via the imposition of a kinetic constraint that produces dynamic torques at each moving joint such that they are a linear function of a single motor command. The linear relationship between dynamic torques at each joint has been demonstrated for multijoint upper limb movements. The purpose of the current study was to test the applicability of such a control scheme to the unconstrained portion of the gait cycle – the swing phase.

**Methods:**

Twenty-eight neurologically normal individuals walked along a track at three different speeds. Angular displacements and dynamic torques produced at each of the three lower limb joints (hip, knee and ankle) were calculated from segmental position data recorded during each trial. We employed principal component (PC) analysis to determine (1) the similarity of kinematic and kinetic time series at the ankle, knee and hip during the swing phase of gait, and (2) the effect of walking speed on the range of joint displacement and torque.

**Results:**

The angular displacements of the three joints were accounted for by two PCs during the swing phase (Variance accounted for – PC1: 75.1 ± 1.4%, PC2: 23.2 ± 1.3%), whereas the dynamic joint torques were described by a single PC (Variance accounted for – PC1: 93.8 ± 0.9%). Increases in walking speed were associated with increases in the range of motion and magnitude of torque at each joint although the ratio describing the relative magnitude of torque at each joint remained constant.

**Conclusion:**

Our results support the idea that the control of leg swing during gait is simplified in two ways: (1) the pattern of dynamic torque at each lower limb joint is produced by appropriately scaling a single motor command and (2) the magnitude of dynamic torque at all three joints can be specified with knowledge of the magnitude of torque at a single joint. Walking speed could therefore be altered by modifying a single value related to the magnitude of torque at one joint.

## Background

Since walking is an essential component of human mobility, the manner in which it is controlled by the central nervous system (CNS) is a fundamental issue in the study of human motion [1-5]. Walking also represents a complex control problem in which the activation of many muscles must be coordinated such that many body segments are rotated about their joints in a manner that maintains balance and ensures a smooth gait. Numerous studies have provided a detailed description of the gait cycle [6,7], and the changes that occur when walking speed is modified [8,9]. The majority of these studies describe the patterns of joint displacement or torque at individual joints during the gait cycle [e.g. [10-12]] and the changes that occur when walking speed is modified [9,12-17]. These studies however, do not address two questions that deal, respectively, with the complexity and generalization of control. The first question is: Are there rules that describe the relationships among multiple joints such as the ankle, knee and hip during locomotion since successful locomotion requires the coordination of all three joints and their associated muscle groups? The second is: Do features of the gait cycle that are conserved across different speeds provide insight into how speed is intentionally changed [18,19]?

Many authors have suggested that for movements that involve multiple body segments, kinematic descriptions of the moving segments or joints may be reduced to a small number of variables. For example, it has been proposed that upper limb reaching movements are controlled in such a way that a rectilinear path is followed by the hand [20]. It has also been demonstrated that this invariant characteristic is unaffected by the speed at which the reaching movement is performed [21]. For walking, it has been demonstrated by a number of authors that the angular displacements of the three lower limb segments (thigh, shank, foot) in the sagittal plane can be accurately described as a combination of two variables [22-24]. Mah et al. [24] also demonstrated that when the motion of both legs is considered, and foot rotations included about two axes (eight angles in total), the segmental rotations are well described by three variables. The idea that a kinematic rule of inter-joint coordination is used in the control of gait receives additional support from evidence that the number and shape of variables required to describe the set of segment rotations are almost identical during normal walking and when kinematic perturbations are imposed such as joint bracing [24], obstacle avoidance [24], or a curved walking trajectory [25]. These results imply that a certain amount of adaptability is possible during walking by rescaling a single set of kinematic variables.

Other investigators have argued that rules for coordination are not kinematic but kinetic. This argument is based on the fact that neural excitation gives rise to muscle forces, and that movement kinematics are consequences of muscle forces. As such, there should be higher correlations between kinetic measures at each joint (computed from the motion of limb segments with inverse dynamic equations) than from kinematic measures. For example, Gottlieb and colleagues [26] have shown that there is a linear relationship between shoulder torque and elbow torque for movements of different speeds and loads, and they have referred to this relationship as "linear synergy". This relationship is established before the end of the first year of life [27]. Winter [4] has also suggested that a compensatory relationship between the hip, knee and ankle moments may exist such that the time series corresponding to their sum is held invariant across walking speeds, despite speed-dependent changes in the time series at each joint. Ivanenko et al. [28] have also provided evidence that a small number of electromyographic variables are capable of accounting for the patterns of muscle activity across the gait cycle during walking. While these studies propose kinetic or electromyographic rules for inter-joint coordination, none of them directly compare how well a kinematic relationship would account for the data.

In the current study we followed a similar methodology to that used by Thomas, Corcos and Hasan [29], who demonstrated considerable reductions in the dimensionality of kinematic and kinetic data obtained from a whole body movement. We applied PC analysis to both kinematic and kinetic data from the swing phase of gait in order to determine the extent to which the dimensionality of each data set could be reduced. The first hypothesis was that more than one PC would be required to account for the variance in angular displacement at the ankle, knee and hip during the swing phase of comfortable walking. This hypothesis was based on the observation that there is high within and between trial variability in joint angular displacement during gait [18]. It was also based on our prior observations that there is a low correlation between kinematic measures of individual joints and gait speed [10]. The second hypothesis was that linear synergy (defined as a case in which a single PC is sufficient to describe the variance in muscle torques across multiple joints) would be present across the ankle, knee and hip for the swing phase of comfortable walking in the sagittal plane. The hypothesis that one PC would account for the variance in joint torques during the swing phase was based on prior studies of unconstrained upper limb movements [26]. It was also based on prior observations in our laboratory that there is a strong linear or quadratic relationship between kinetic measures of individual joints and gait speed [10]. In this study a linear relationship was demonstrated between the peak hip flexion moment and walking speed during the swing phase and a quadratic was demonstrated during the stance phase. The third hypothesis was that the variance accounted for by the first PC would not change with movement speed. This hypothesis was based on the observation that the shape of ankle, knee and hip torque time series remain similar across movement speed [8]. The fourth hypothesis was that the magnitude of the dynamic joint torques would increase with speed during the swing phase, and that a single PC would sufficiently account for the variance. This hypothesis was based on previous studies that have shown that increased walking speed is associated with increased torques at all three joints [11]. We also hypothesized that the magnitude scaling would be proportional amongst the three joints [29].

## Methods

### Protocol

Twenty-eight healthy, able-bodied adults participated in this study. All participants were in good health with no known neurological, orthopedic or cardiopulmonary diagnoses. The twenty-eight participants (14 female and 14 male) were 20–34 years old (mean 26.0 years), 155.5–191.5 cm in height (mean 169.2 cm), and weighed 44.5–85.5 kg (mean 66.3 kg). The study was approved by the Spaulding Rehabilitation Hospital Internal Review Board and informed consent was obtained from all participants prior to participation.

Each participant completed one testing session in which biomechanical data were collected while walking barefoot at three walking speeds over a distance of 10 meters. Each participant was first asked to walk at his/her own self-selected comfortable pace. Participants were timed with a stop watch. Participants were then asked to walk 25% faster than the comfortable pace and then at 25% slower than the comfortable pace. Feedback, based on the stopwatch time, was given to participants after each trial as to whether they walked too fast or slow and only trials completed at the required pace were retained for further analysis.

### Experimental set-up and procedures

An eight camera video-based motion analysis system (Vicon, Oxford Metrics, Oxford, UK) was used to measure the three-dimensional position of markers attached to the following bony landmarks: anterior superior iliac spine, posterior superior iliac spine, lateral femoral condyle, lateral malleolus, forefoot and heel. Additional markers were rigidly attached to wands over the mid-femur and mid-tibia. The following anthropometric measurements were also recorded: body weight, height, and leg length measured from the medial malleolus to the anterior superior iliac spine, knee width, and ankle width.

Participants walked barefoot at each of the three walking speeds across a ten-meter walkway. Motion data were collected synchronously with data from two staggered force platforms (AMTI, Watertown, MA) embedded in the walkway in order to obtain ground reaction forces and torques. Data were collected at a rate of 120 frames per second. For each condition, four trials with acceptably continuous marker data (those with no discontinuities caused by obscured markers or extraneous light sources) and ground reaction force data were retained for further analysis. In each trial, a single swing phase was retained for analysis. The instant of first foot contact (as indicated by the onset of ground reaction force) served as a landmark to separate the end of the swing phase from the beginning of the stance phase and the first data point following the cessation of force application on the force plate designated the beginning of the swing phase.

Joint angular displacements were derived using an Euler angle sequence in which the primary rotation angle was defined as a rotation about a medial-lateral axis (i.e. flexion-extension angles). While gait activities clearly involve joint rotations about an anterior-posterior axis (abduction-adduction) and a vertical axis (medial-lateral rotation), we chose to focus our analyses on the joint angular displacements corresponding to flexion and extension of the hip, knee, and ankle (dorsiflexion of the ankle is referred to as flexion throughout this paper, and plantarflexion as ankle extension). Anthropometric characteristics [measured and derived from Dempster's [30] data], derived linear and angular velocity, accelerations of the lower limb, and joint center position estimates were used to compute internal joint torques using a modified version of a commercially available full-inverse dynamic model (Vicon Bodybuilder, Oxford Metrics, Oxford, UK). The focus of this paper is on the transient pulses of torque that propel and arrest the limb. On these are superimposed the static torque requirements for resisting gravity. We assumed the separability of the two components, a static one proportional to gravity and a dynamic one independent of it. The modified version of the inverse dynamic model removed the gravitational component from the joint torques [26]. As with the kinematic data, we report the dynamic joint torques (also referred to elsewhere as muscle torques or net muscle torques) corresponding to flexion and extension of the right hip, knee, and ankle. Joint torque was normalized to body weight and reported as internal torque in Newton-meters per kilogram.

### Principal component analysis

Principal component analysis was used in order to determine the extent to which the observed patterns of joint angular displacement and dynamic torque could be described by a data set of fewer dimensions. The appropriate use of PC analysis with biological data of this type has been demonstrated previously by a number of authors [22,31,32]. Two types of PC analysis were applied to each data set (i.e. angular displacement and dynamic torque data sets). First, the time series data were analyzed in order to determine the similarity in shape of each time series. Second, the peak-to-peak range of each time series was analyzed to determine whether a linear relationship existed in the scaling of kinematic and kinetic data across participants and speeds.

When analyzing the time series data, the input array consisted of data from all participants, all trials and from each of the three joints. The resulting input data set (either joint angles or torques) therefore consisted of 336 time series (28 participants × 3 joints × 4 trials), each 101 points in length. Each time series was normalized by subtracting the mean and subsequently dividing each value by the standard deviation of the series. The PCs were determined using the *princomp *function in Matlab (Statistics toolbox 5.0, Mathworks, Waltham, MA). This procedure is equivalent to calculating the PCs based on the correlation matrix of the input data. Calculating the PCs in this way standardizes the variance of each time series at one, thereby removing possibility that time series that vary over a large range dominate the first few PCs. The output of each PC analysis consisted of 336 PCs, each 101 points in length. The variance accounted for by each PC was used as the criterion upon which the retention or rejection of PCs was based. The variance accounted for by each PC was assessed by analysis of the corresponding eigenvalue. Only PCs associated with an eigenvalue of at least 1 were retained for further analysis [33]. Given the fact that time series from each joint in every trial were included in the PC analysis, this represents a very conservative approach to the selection of the PCs. Projections of the data onto each PC were derived as the product of the PC eigenvector and the associated raw data. These projections assist in the visual interpretation of each PC and are henceforth referred to as eigencurves [29]. Each eigencurve was normalized to its peak-to-peak range. The interpretation of each eigencurve was facilitated by examination of the values within the eigenvector associated with each joint (joint loadings). In this paper we present the mean of the absolute joint loadings across participants and walking speeds. Absolute values were taken for each loading since the assignation of positive and negative values is an arbitrary choice made during the calculation of principal components and retaining the assigned polarity may alter the results of averaging. The loading value at each joint, relative to those at the other joints, reflects the extent to which the pattern described by the associated eigencurve is present within the data for that joint. For example, a large loading at the knee joint relative to those at the hip and ankle would indicate that the pattern of data (angular displacement or torque) at the knee is well described by the eigencurve under consideration, whereas the patterns at the other joints are not well described by the same eigencurve.

When analyzing the range of angular displacement and torque at each joint, the data set (either joint angle range or dynamic torque range) consisted of 3 columns (one for each joint) by 336 rows (28 participants × 3 speeds × 4 trials). The PC analysis was performed using a covariance matrix, a method that retains information regarding the relative magnitude of each variable. The method for calculating the PCs was identical to that used previously (i.e. the mean of the data is subtracted prior to the PC analysis) with the exception that the data was not divided by its standard deviation. The output of this PC analysis consisted of 3 PCs, each 336 points in length. In this case, a single PC that accounts for all of the task-important variance in a data set suggests that the data lie nearly on a straight line in three-dimensional space and the three magnitudes are determined by a single variable. When analyzing joint torques for example, this would imply that the magnitude of torque at one joint determines the magnitude of torque at each of the remaining joints. Furthermore, if the PC vector passes through the origin of the three-dimensional space, it would imply that the magnitude of torque at one joint is always directly proportional to the magnitude of torque at each other joint [29]. The same logic applies when analyzing joint angular displacements.

## Results

### Sagittal plane kinematics

The walking speeds of men and women were not significantly different and differed by less than 1% at each speed. Therefore we present mean values for the entire sample, shown in Table 1. Consistent with the instructions given to participants, the average walking speed increased from a mean of 1 m/s in the 'slow' walking condition to 1.87 m/s in the 'fast' condition. In agreement with previous studies [9,12,13,16], many temporal parameters changed with walking speed. Cadence increased, the stance phase as a percentage of the gait cycle decreased by 4.2% as walking speed increased, as did the duration of the entire gait cycle. Increases in walking speed were also associated with increases in both step and stride length.

**Table 1:** Descriptive measures of gait derived from kinematic data for all walking speeds [mean (SD)]

	**Slow**	**Comfortable**	**Fast**
Walking Speed [m/s]	1.00 (0.16)	1.32 (0.14)	1.87 (0.21)
Cadence [steps/min]	101 (10.8)	118 (8.7)	141 (15.7)
Duration of Stance [%]	62.7 (2.0)	60.7 (1.6)	58.5 (1.4)
Gait Cycle Duration [s]	1.20 (0.14)	1.02 (0.08)	0.86 (0.09)
Step Length [m]	0.59 (0.07)	0.67 (0.07)	0.79 (0.9)
Stride Length [m]	1.18 (0.14)	1.35 (0.14)	1.59 (0.18)

**Figure 1:**
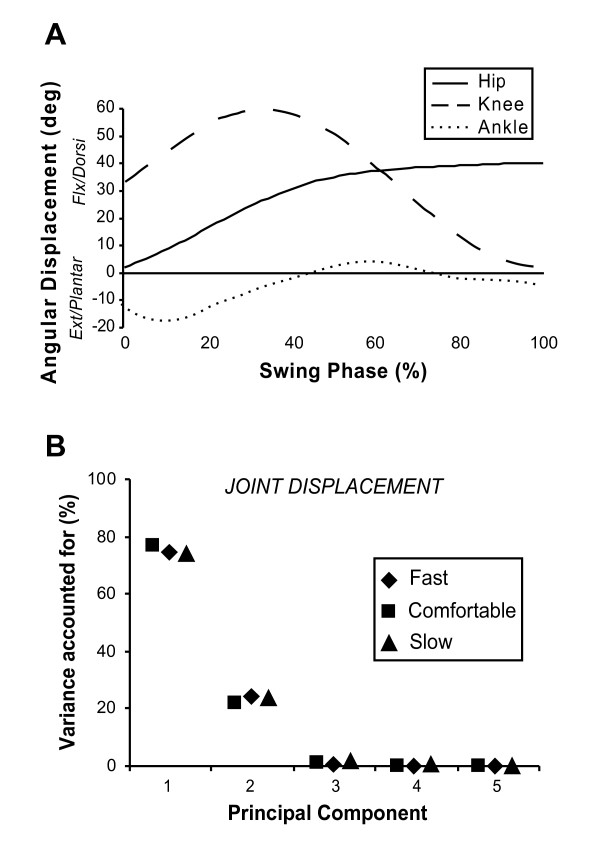
**Angular displacement at each joint**. **A**) Average sagittal plane angular displacement time series for the hip (solid), knee (dashed), and ankle (dotted) for a representative participant walking at comfortable speed. **B**) The percentage of total variance accounted for (VAF) in joint angular displacement by each of the first five PCs. Results are shown for comfortable (square symbols), fast (diamond symbols), and slow (triangular symbols) walking speeds during the swing phase.

The data in Figure 1A present swing phase angular displacement data averaged over four trials from one representative subject for the hip, knee and ankle for the comfortable speed condition. The data show that the hip initially flexes to approximately 30° and maintains a similar angular position from that point through to heel contact. The knee flexes to a peak of 60° at around 30% of the swing phase and straightens again prior to heel contact. The ankle reaches 20° of plantarflexion just after foot-off before dorsiflexing during swing until just prior to foot contact where plantarflexion ensues. Visual inspection of the three excursions suggests that the time at which flexion changes to extension is different across the three joints.

The first PC associated with joint angular displacement accounted for an average of **75.1% **(SD = 1.4) of the variance during the swing phase (Figure 1B). A second PC accounted for **23.2% **(SD = 1.3) of the variance. These data indicate that the patterns of angular displacement for the three joints across all trials can be well described as a combination of two time series during the swing phase. The variance accounted for by each PC was extremely consistent across the three walking speeds.

### Sagittal plane kinetics

**Figure 2:**
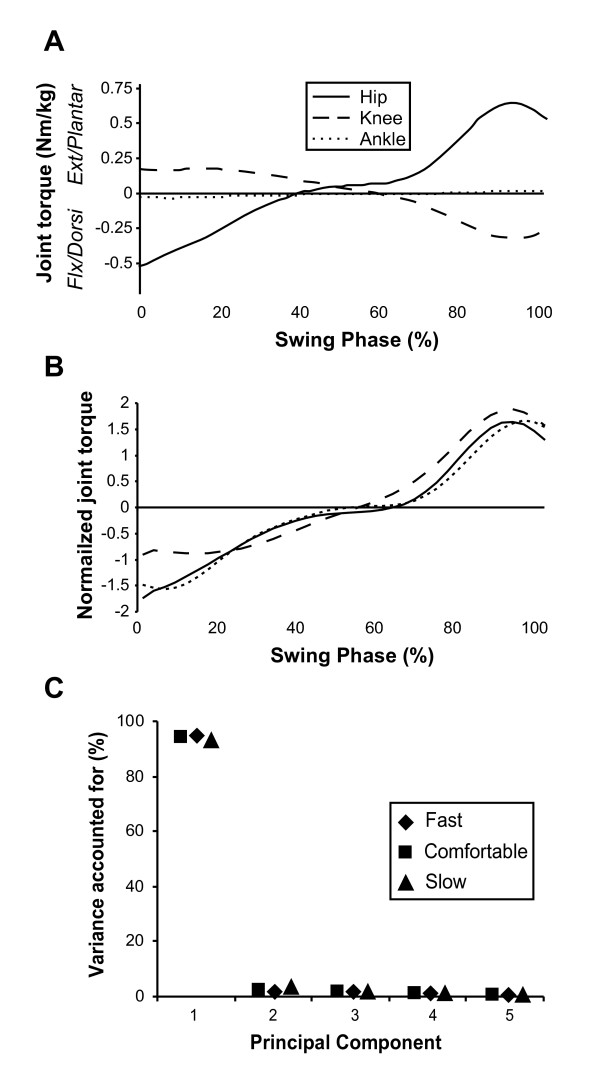
**Dynamic joint torque at each joint**. **A**) Average sagittal plane dynamic joint torque time series for the hip (solid), knee (dashed), and ankle (dotted) for the same participant walking at a comfortable speed. Time series in these plots are shown in degrees from 0–100% of the swing phase. Positive values signify flexion or dorsiflexion while negative values signify extension or plantarflexion. **B**) Swing phase joint torques are presented, having been normalized such that the variance across each time series is equal to one. The knee torque data was also inverted by multiplying raw data by -1. **C**) The percentage of total variance accounted for (VAF) in dynamic joint torque by each of the first five PCs. Results are shown for comfortable (square symbols), fast (diamond symbols), and slow (triangular symbols) walking speeds during the swing phase.

The data in Figure 2A present dynamic joint torque traces for the hip, knee and ankle averaged over four trials for one representative participant in the comfortable speed condition. The data show that after initiating the swing phase with flexion, the hip torque then reverses to a maximum extension torque of 0.7 Nm/kg at around 90% of the swing phase. On the contrary, the knee torque begins the swing phase in extension before reaching a peak flexion torque of about 0.3 Nm/kg at 90% of the swing phase. Although relatively small throughout the swing phase, the ankle torque begins in dorsiflexion before making a transition to plantarflexion during mid-swing and reaching a maximum plantarflexion at around 90% of the swing phase. This pattern is clearly visible in Figure 2B in which the data have been normalized such that the variance of each time series is equal to one. Visual comparison of the shapes of the joint torque time series suggests a similarity within joints during the swing phase (most clearly illustrated in Figure 2B).

**Figure 3:**
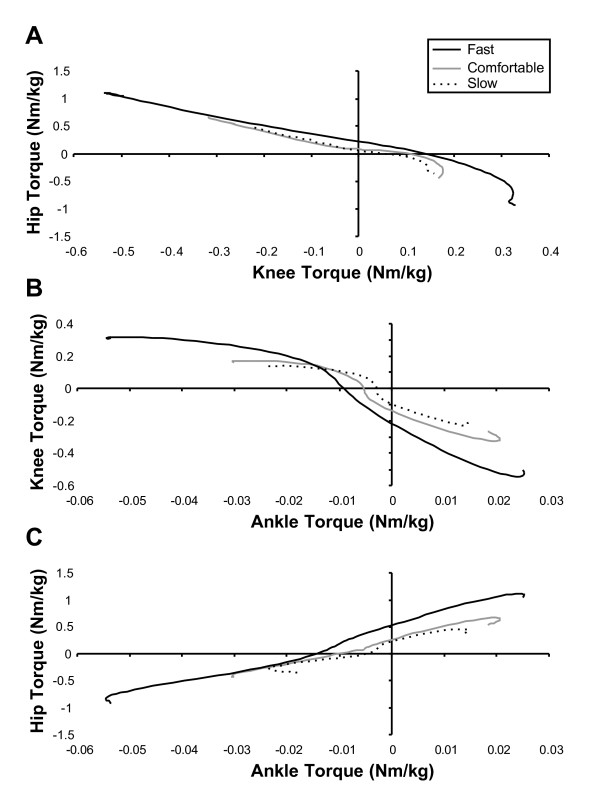
**Hip, knee and ankle torque/torque plots**. Sagittal hip vs. knee (**A**), knee vs. ankle (**B**), and hip vs. ankle (**C**) joint torque comparison for a representative participant walking at comfortable (solid black), fast (solid gray), and slow (dotted) speeds. These plots show the linear relationship between pairs of joints during the swing phase.

The results of the PC analysis showed that a single PC accounted for an average of **93.8% **(SD = 0.9) of the variance during the swing phase. The variance accounted for by the first PC was once again remarkably consistent across speeds in each phase as can be seen in figure 2C. The fact that a single PC accounted for such a large proportion of the variance in joint torques during the swing phase indicates that the torque produced during the swing phase follows an essentially identical pattern at each joint and in each trial. The existence of a linear torque relationship is further highlighted in Figure 3, in which joint torques at the hip, knee and ankle are plotted against one another. It is evident from these plots that the relationships established between joints remain stable across walking speeds, despite changes in the magnitude of the torques produced.

### Eigencurves and loadings

Eigencurves (projections of the original data onto each retained PC) provide a representation of each PC that allows us to consider their functional relevance in the context of the task. In this task set, the eigencurves also allow us to observe the impact of changes in walking speed upon the emergent patterns of joint angular displacement and torque production. Eigencurves are presented for each of the PCs retained following an analysis of the amount of variance accounted for by each (see methods). If a joint loads heavily onto a particular PC, it can be said that the shape of the associated eigencurve reflects an important pattern in the data produced at that joint. The joint loadings can therefore be used to interpret the functions associated with each PC.

#### Kinematic eigencurves

**Figure 4:**
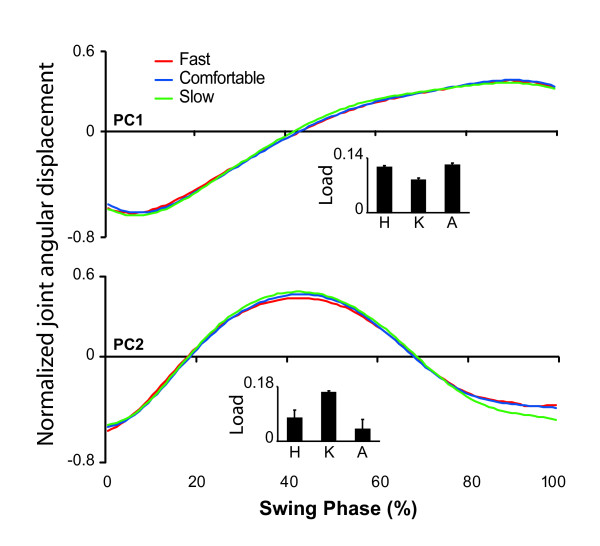
**Kinematic eigencurves**. Eigencurves for each retained kinematic PC are shown for fast (red), comfortable (blue), and slow (green) walking. All eigencurves were normalized by their maximum peak to peak range and are therefore presented in arbitrary units. Joint loadings on each PC (mean + SD across walking speeds) are inset and are located with the eigencurve with which they are associated.

The eigencurve associated with the first swing phase PC makes a single, smooth transition from an initial low value to a higher value (Figure 4 – PC1). All joints load onto this PC to approximately the same extent (Figure 4 – see PC1 inset), suggesting that this represents the basic requirement to move the joints into a position that prepares the leg for foot contact and the absorption of weight. The second PC peaks after 40% of the swing phase, suggesting that this movement may be related to ensuring that the foot avoids striking the ground mid-swing (Figure 4 – PC2). This PC primarily reflects angular motion at the knee joint with some motion also at the ankle (Figure 4 – see PC2 inset). Interestingly, each eigencurve was extremely consistent in shape across the three walking speeds.

#### Kinetic eigencurves

**Figure 5:**
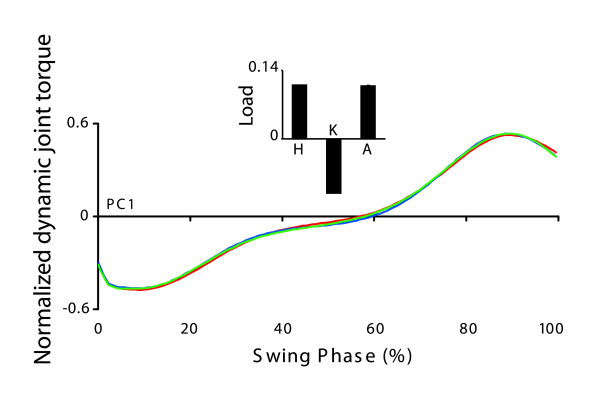
**Kinetic eigencurve**. Eigencurves for the single retained kinetic PC are shown for fast (red), comfortable (blue), and slow (green) walking. All eigencurves were normalized by their maximum peak to peak range and are therefore presented in arbitrary units. Joint loadings on each PC (mean + SD across walking speeds) are inset and are located with the eigencurve with which they are associated.

A single kinetic PC was sufficient to account for the variance in dynamic joint torques at the three joints during the swing phase in all trials (Figure 5). The implication of this result is that not only is the fundamental pattern of torque production identical at each joint, but also from trial to trial. It is not surprising that, given the fact that a single PC was required in this case, that each of the three joints load onto the PC with approximately equal weights (Figure 5 inset). This simply demonstrates that each joint was following the same pattern of torque production as is reproduced by the eigencurve. The remarkable feature of this data however, is the fact that torque production at each of the three joints proceeds in an essentially identical manner despite changes in the magnitude of torque at each joint and changes in magnitude that mirror those in walking speed.

### Ranges of joint angular displacement and joint torque

**Figure 6:**
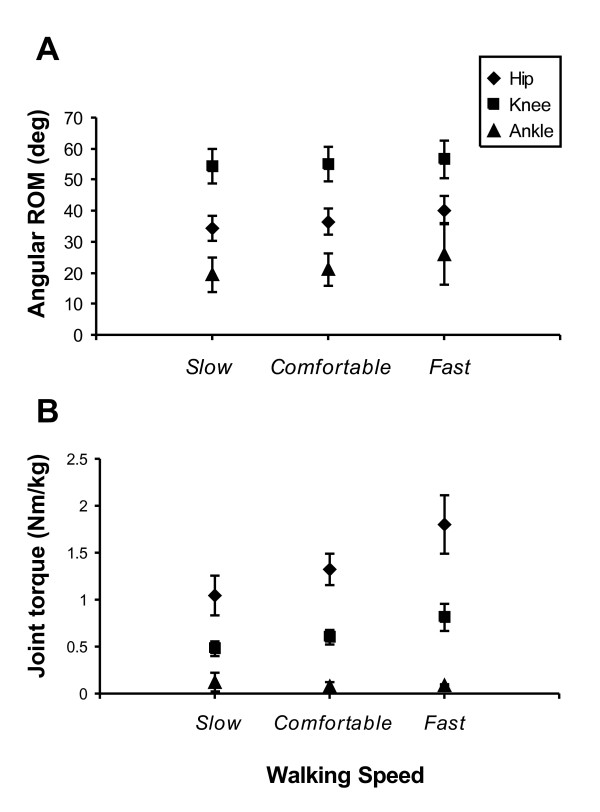
**Range of angular displacement and torque at each walking speed**. Effect of speed on sagittal joint angular displacement (**A**) and dynamic joint torques (**B**) for the hip (diamond), knee (square), and ankle (triangle). Mean data and standard deviations for all participants are plotted for slow, comfortable, and fast walking speeds. Speed had a statistically significant effect on all parameters plotted.

The consistency of each eigencurve presented indicates that the fundamental patterns of joint displacement and torque production are invariant across walking speeds. Questions remain however, as to the manner in which walking speed is intentionally modified and whether a single coordinative rule can be identified that describes the changes at each joint that are associated with speed modulation. The data in Figure 6A show the effect of speed on the range of angular displacement at each joint during the swing phase. Increases in speed were associated with statistically significant increases in the range of angular displacement at the hip (*F*[2,27] = 56.4, *p *< 0.0001) knee (*F*[2,27] = 5.3, *p *= 0.0082) and ankle (*F*[2,27] = 15, *p *< 0.0001). The maximum peak-to-peak torque at each joint was also shown to increase with increases in walking speed at the hip and knee during swing (Hip: *F*[2,27] = 187.6, *p *< 0.0001; Knee: *F*[2,27] = 190.3, *p *< 0.0001) (Figure 6B). The range of torque at the ankle during the swing phase decreased significantly as walking speed increased (*F*[2,27] = 3.6, *p *= 0.034), although the absolute change was quite small (Figure 6B). These data collectively show that increased walking speed is accompanied by increases in the range of angular displacement at each joint and increases in peak-to-peak torque at the hip and knee.

While PC analyses of the time series kinematic and kinetic time series gave insight into the commonalities within the shapes of these time series, we sought to identify whether the scaling of angular displacement or dynamic torque at each joint could be described by a linear relationship. For the range of angular displacement, a single principal component accounted for **63.3% **of the variance during the swing phase. This result indicates that the ranges of angular displacement at each joint are not related by a simple linear constraint. For the dynamic joint torques, one principal component accounted for **99.3% **of the total variance in the swing phase, indicating that the relationship between the peak-to-peak torque ranges at each joint is linear.

**Figure 7:**
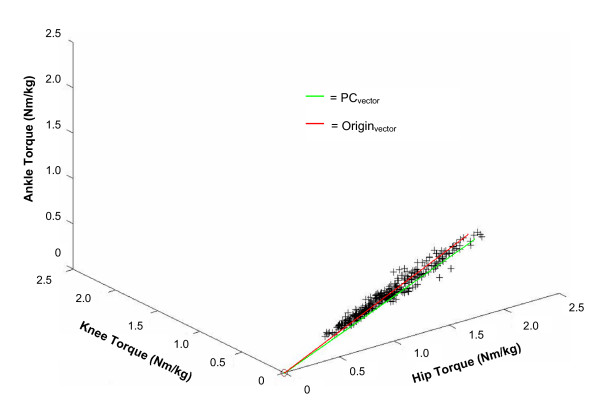
**Relationship between the range of dynamic torque at each joint**. The magnitudes of dynamic hip, knee, and ankle joint swing phase torques from all 336 trials are plotted. In addition, the PC vector (green) formed by the 1^st ^PC and the Origin vector (red), going from the origin of this three-dimensional joint space to the mean magnitude of dynamic hip, knee, and ankle torques, are shown. The angle between these two vectors is small, measuring 3.9°, which indicates that the ratios between the torques at each joint are not altered with changes in walking speed.

In Figure 7 the peak-to-peak magnitudes of joint torque of the ankle, knee and hip during the swing phase from all trials are plotted and the vector formed by the first PC is shown. We also illustrate the vector from the origin of this space to the mean peak-to-peak dynamic joint torques of the ankle, knee and hip. In order to determine if there is a proportional relationship between the magnitudes of the joint torques, the angle between the PC1 vector and the line joining the origin to the mean point was determined. This angle measures **3.9 **degrees. This indicates that PC1 passes very close to the origin of this space. As discussed in Thomas and colleagues [29], this suggests that the magnitudes of torque produced at each joint scale with respect to each other.

## Discussion

Gait is a complicated motor coordination task that involves the coordination of numerous joints, as well as the coordination of both the left and right sides of the body. The data set we analyzed in this study is typical for normal healthy individuals [10-12,34,35]. As reported in many other studies, the participants walked within a range of speeds considered to be indicative of normal gait (0.6 – 2.2 m/s). Several gait parameters changed with speed: cadence, step length and stride length all increased with increasing walking speed, while gait cycle duration and the duration of stance decreased with increasing walking speed. Four findings emerged. First, we found that two PCs are required to describe the relationship between the three joint angles during the swing phase. Second, we have shown that our previous findings of linear synergy (defined as a linear relationship between dynamic torques produced at each moving joint) at the elbow and shoulder [36] extend to the joint torques about the ankle, knee and hip during the swing phase of gait in the sagittal plane. Third, we demonstrated that there is no effect of speed on the shape of emergent principal components. Fourth, we find that the magnitudes of torque at the hip, knee and ankle joints during the swing phase scale linearly and proportionally with increases in speed.

### Kinematics during the swing phase

In the present study, we evaluated the intersegmental coordination between angular displacements of the joints of the lower limb in the sagittal plane during the swing phase of gait. The joint angular displacement data obtained from three joints were accurately described by two PCs, each identifiable in terms of its functional outcome. The first PC described the smooth transition of the joints from their initial orientations following foot-off to their terminal swing phase orientation at foot contact. This type of motion was evident in the angular displacement time series at each of the three joints, as demonstrated by the fact that the first PC loaded to approximately the same extent onto each joint. The second PC was primarily associated with flexion of the knee during mid-swing. The timing of this motion, and its association with the knee joint, makes it likely that this PC describes joint motion that ensures that the foot remains clear of the walking surface during the swing phase. The identification of a kinematic function concerned with ground clearance is consistent with the conclusions of Winter [11] who suggested that the planning of a foot trajectory that ensured ground clearance is one of three primary subtasks essential for the production of normal gait. The fact that two kinematic PCs were identified during the swing phase is also consistent with idea that joint angles are essentially associated by a planar relationship in which two specified variables are sufficient to describe motion at three joints [22-24]. It has previously been demonstrated that the nature of this planar relationship is not altered by perturbing gait patterns [24] and indeed, the relationship between joint angular displacements identified in the current study was robust despite changes in walking speed.

### Linear synergy during the swing phase of gait

The data that we have presented show that the shape of the dynamic torque time series is very similar during the swing phase of gait across all three joints, and that this is independent of speed (Figure 5). The common kinetic time series underlying the swing phase of gait suggested by these data could potentially simplify the control of the swing phase in a manner previously suggested by Gottlieb et al. [36]. Their suggestion, referred to as 'linear synergy', proposes that a single command is generated by the CNS and distributed to task-relevant joints in order to control the pattern of torque production (torques would be scaled in order to appropriately manage the dynamic characteristics of each limb segment). This idea was based upon an upper limb model and restricted to unconstrained motion. In the gait cycle, the swing phase represents the most loosely constrained motion and our finding that a single kinetic time series describes the pattern of torque production at each joint during this phase is therefore consistent with the idea of linear synergy. The small angular offset between the first PC calculated from the torque range data and a vector extending through the mean of the same data set from the origin of the coordinate space (Figure 7) suggests that the control of dynamic joint torques may be further simplified. Specifically, this result demonstrates that the magnitude of torque at all three joints can be predicted by the magnitude of torque at any single joint, regardless of walking speed. Taken together the existence of a single kinetic pattern across joints and the proportional scaling of joint torques support the idea that the swing phase of gait can be controlled via a relatively simple process. Once the pattern and magnitude of torque is specified for one joint, both variables are automatically specified for the remaining joints without further calculation. This may have clinical implications since a gait pattern performed at one speed (e.g. slow) can be generalized to different (e.g. higher) walking speeds without further therapeutic intervention. It remains an open question whether modifications made to the gait pattern of an individual via clinical intervention can be generalized in a similar manner across walking speeds, although previous investigations into the generalization of acquired skill suggest that this may be possible [37,38].

## Conclusion

During the swing phase of gait, at any given speed, walking capitalizes on the fact that dynamic torques generated about all three joints are tightly coupled. A tight coupling of joint torques has now been demonstrated for upper limb movements in adults [36,39], children [27], in upper limb movement reaching tasks that require the maintenance of balance [29], and now in the swing phase of gait. As such, the linear covariation of joint torques appears to be a robust finding that applies not only to unconstrained upper limb movements that are primarily under direct cortical control [36,40,41], but also to the swing phase of locomotion that is under cortical control mediated via spinal and interneuronal networks [42]. Control of the swing phase may be further simplified by the maintenance of a linear and proportional relationship between the range of torque produced at the hip, knee and ankle joints across speeds.

## Authors' contributions

JS drafted the manuscript, JJ was instrumental in acquiring the data and performing data analysis, VP performed data analysis, GLG conceived of the study, JST facilitated interpretation of the PC analyses and DMC participated in the design of the study, coordinated its completion and provided assistance with the drafting of the manuscript.
